# Quantitative MRI in the assessment of clival invasion in locally advanced nasopharyngeal carcinoma

**DOI:** 10.3389/fonc.2026.1751817

**Published:** 2026-08-27

**Authors:** Yuqing Lv, Fayi Yu, Xunmei Liu, Yifan Xu, Min Hu, Lulu Huang, Rensheng Wang, Tingting Zhang

**Affiliations:** 1 Department of Radiation Oncology, The First Affiliated Hospital of Guangxi Medical University, Nanning, Guangxi, China; 2 Key Laboratory of Early Prevention and Treatment for Regional High-Frequency Tumors (Guangxi Medical University), Ministry of Education, Nanning, Guangxi, China; 3 Department of Radiology, The First Affiliated Hospital of Guangxi Medical University, Nanning, Guangxi, China

**Keywords:** clival invasion, imaging biomarker, nasopharyngeal carcinoma, quantitative magnetic resonance imaging, radiotherapy

## Abstract

**Objective:**

This study assesses the value of quantitative magnetic resonance imaging (qMRI) in evaluating clival invasion (CI) and monitoring therapeutic response in locally advanced nasopharyngeal carcinoma (LA-NPC). It also investigates the relationship between qMRI parameter changes in CI and regression of nasopharyngeal soft-tissue invasion.

**Methods:**

In this prospective study, fifty-five LA-NPC patients with CI from a single-center underwent qMRI at baseline (t0), pre-radiotherapy (t1), mid-radiotherapy (t2), and post-radiotherapy (t3). T1, T2, and T2* mappings were measured in manually delineated volumes of interest. Patients were categorized into complete response (CR) and non-CR groups based on nasopharyngeal soft tissue tumor regression per RECIST 1.1. The cervical vertebrae served as self-controls.

**Results:**

At baseline, T1, T2, and T2* mapping values were significantly higher in clival invasion than in normal cervical vertebrae (*p* < 0.001). During treatment, the CR group (87.3%) exhibited marked decreases in T1 values (from 841.30 [IQR:521.31] ms at baseline to 587.61 [IQR: 187.54] ms at post-RT; p<0.001) and T2* values (from 7.27 [IQR: 5.28] ms to 4.86 [IQR: 3.48] ms; p<0.001), accompanied by a significant increase in T2 values (from 89.83 [IQR: 18.25] ms to 99.79 [IQR: 12.10] ms; p<0.001), whereas the non-CR group showed no significant alterations in T1 and T2 mapping values (*p* > 0.05). Notably, there were no significant differences in T1, T2, and T2* mapping values between t2 and t3 in the CR group (*p* > 0.05). Furthermore, differences between CI and cervical vertebrae measurements (ΔT1, ΔT2, ΔT2*) diminished in the CR group during treatment (*p* < 0.05). Conversely, the non-CR group showed no significant changes in ΔT1 (*p* = 0.738) mapping values but did exhibit changes in ΔT2 and ΔT2* mapping values (*p* < 0.001).

**Conclusions:**

The qMRI-derived T1, T2, and T2* mapping demonstrates potential utility in monitoring CI response in LA-NPC, serving as an inherently quantitative tool that warrants further investigation in larger cohorts.

## Introduction

Nasopharyngeal carcinoma (NPC) is an epithelial malignancy with a pronounced geographical distribution, exemplified by China constituting 42.4% of the global 120,416 new cases reported in 2022 (1, 2). Due to its concealed anatomical location, over 70% of NPC cases are initially diagnosed at a locally advanced stage, frequently complicated by skull base invasion—a key prognostic factor strongly associated with higher local recurrence rates and poorer survival outcomes (1–4). The clivus is the primary site of involvement, observed in 40.33% to 42.90% of cases (3, 5–7). The clinical management of clival invasion (CI) is inherently dependent on imaging, with magnetic resonance imaging (MRI) serving as the primary tool for diagnosis, staging, therapy monitoring, and follow-up, given the risks associated with direct biopsy (8, 9). However, conventional MRI faces significant limitations in the post-treatment setting. Fundamentally qualitative, visual assessment of tumor regression is often subjective and prone to considerable interobserver variability. Furthermore, distinguishing true tumor persistence from post-radiotherapy reactive changes, such as inflammation, fibrosis, or osteomyelitis, remains a major diagnostic challenge on conventional sequences (10). Although advanced metabolic imaging modalities such as ^18^F-FDG PET/CT and SPECT/CT can detect metabolically active areas, their utility for routine longitudinal monitoring is hindered by high costs, ionizing radiation, and a risk of false- positive findings induced by radiation-related inflammation (11–13). These limitations highlight the need for objective, radiation-free, and quantitatively reliable methods to dynamically assess CI improvement in patients with locally advanced NPC (LA-NPC), thereby supporting decisions regarding subsequent treatment.

Recent advances in imaging technology have led to an increased focus on quantitative evaluation of tumor characteristics. Quantitative MRI (qMRI) has emerged as a promising approach, encompassing various techniques including relaxation rate mapping (T1, T2, and T2* mapping) (14). In the context of bone marrow infiltration, these absolute parameters reflect distinct pathophysiological processes rather than mere visual alterations. T1 mapping measures longitudinal relaxation time, which is highly sensitive to tissue composition shifts, particularly water and fat content (15). T2 mapping measures transverse relaxation time, reflecting proton phase coherence loss and proving valuable in detecting inflammation, edema, and fibrosis (16). T2* mapping measures effective transverse relaxation time, combining T2 relaxation and magnetic field inhomogeneity effects, demonstrating particular sensitivity to magnetic susceptibility differences caused by blood products, iron deposition, or calcifications (17, 18). The employment of quantitative assessment facilitates the precise identification of lesions, thus guiding the development of personalized treatment regimens for patients, with the result that harm caused by inadequate or excessive treatment can be reduced. These parameters represent fundamental intrinsic properties in MRI physics and have been extensively utilized in solid tumor evaluation due to their clinical relevance (19–22). While qMRI has been successfully explored in bone metastases and benign skeletal lesions (19, 23), its specific role in monitoring the unique bone marrow remodeling of CI in LA-NPC remains unexplored. Moreover, bony structural regression is typically delayed and highly challenging to quantify using conventional anatomical metrics. Therefore, to validate qMRI as a clinically relevant surrogate marker, it is essential to establish how these intramedullary changes correlate with the regression of the primary nasopharyngeal soft-tissue tumor, which serves as the established clinical benchmark according to RECIST 1.1 (24). To this end, the present study aimed to evaluate the feasibility of using qMRI to assess CI in LA-NPC, systematically characterizing the longitudinal relationship between clival parametric alterations and macroscopic soft-tissue tumor regression.

## Methods

### Patients and grouping

Approved by the institutional review board of the First Affiliated Hospital of Guangxi Medical University (study number: 2023-E531-01), this study prospectively enrolled patients with histologically confirmed NPC between January 2023 and August 2024. All patients were staged according to the 8^th^ edition of the American Joint Committee on Cancer (AJCC) staging system. Inclusion criteria comprised: Age between 18 and 70 years; No prior tumor-related treatment before MRI examination; Clival invasion; Complete clinical data availability. Exclusion criteria included: Prior radiotherapy to the head and neck region; MRI contraindications such as cardiac pacemakers or cochlear implants; Inability to complete the full course of radiotherapy; Geometric distortion or physiologic motion artifacts on MR images; The cervical vertebra invaded or other cervical disorders. CI was diagnosed based on the following MRI criteria: (1) a defect in the low-signal-intensity cortex on T1-weighted images, (2) replacement of high-signal-intensity marrow with low-signal-intensity tissue on T1-weighted images, and (3) the abnormal tissue described in (1) and (2) exhibiting moderate contrast enhancement on post-contrast T1-weighted images (3, 25). Additional diagnostic indicators included erosion or sclerosis of the bone cortex on CT, or abnormal signals detected on ^18^F-FDG PET/CT or SPECT/CT. Treatment response was assessed according to RECIST 1.1 based on the primary nasopharyngeal soft-tissue lesion. Because clival invasion represents bone involvement and is considered non-measurable by RECIST 1.1, RECIST criteria were not used to directly evaluate the response of clival bone invasion. Moreover, in routine response assessment for nasopharyngeal carcinoma, treatment efficacy is primarily evaluated based on the regression of measurable soft-tissue lesions. Therefore, RECIST 1.1-based complete response (CR) of the primary soft-tissue tumor was used as a practical reference endpoint for local treatment response, rather than as a direct assessment of the clival invasion itself. Patients were categorized into CR and non-CR groups according to the response status of the primary soft-tissue tumor, and longitudinal qMRI changes in the clival invasion were compared between these groups. All participants provided written informed consent, and the study adhered to the principles of the Declaration of Helsinki and the International Conference on Harmonization of Good Clinical Practice guidelines.

### Treatment

Radiotherapy was delivered in accordance with the International Commission on Radiation Units and Measurements Reports 50 and 62 guidelines. The prescribed doses were as follows: 70–72 Gy for the planning gross target volume of the nasopharynx, 66–70 Gy for involved lymph nodes, 60–64 Gy for high-risk planning clinical target volume, and 54–56 Gy for low-risk planning clinical target volume. Treatment was administered in 30–33 fractions, with five fractions per week. The treatment protocol comprised induction chemotherapy (IC) followed by concurrent chemoradiotherapy (CCRT). All patients received platinum-based chemotherapy (80–100 mg/m²) every three weeks during CCRT, for a maximum of three cycles.

### MRI protocol

All MRI examinations were performed using a 3T scanner (Premier, GE Healthcare, WI) with a 21-channel head and neck phase array coil. Patients were positioned head-first and supine. The imaging protocol included: T1 and T2 mapping sequences using magnetic resonance image compilation (MAGiC) in the axial direction with the following parameters: repetition time (TR): 4000 ms; echo time (TE): 21 ms; flip angle: 120° saturation pulse, a 90° excitation pulse applied after a specific delay time, and a subsequent train of 180° refocusing pulses; bandwidth: 22.73 kHz; slice thickness: 6 mm; field of view (FOV): 260 mm; acquisition matrix: 256 × 256; number of excitations (NEX): 1; scan time: 4 min 32 s. T2* mapping parameters: TR: 52.9 ms, TE: 1.5-14.2 ms; flip angle: 20°; bandwidth: 125.0 kHz; slice thickness: 6 mm; FOV: 180 mm; acquisition matrix: 96 × 96; NEX: 2; scan time: 53 s. All scans were performed without contrast administration. Patients underwent MRI at four predefined time points: baseline (t0), before any treatment; pre-radiotherapy (pre-RT, t1), within 1 week prior to the start of radiotherapy; mid-radiotherapy (mid-RT, t2), after receiving approximately 60 Gy of radiotherapy; and post-radiotherapy (post-RT, t3), within 1 week after completion of radiotherapy.

### Image processing and segmentation

Raw images were processed using SyMRI software (version 8.0, Synthetic MR, Linköping, Sweden) to generate T1 and T2 mappings. T2* mapping was performed using a vendor-provided post-processing workstation (version 4.7). Quantitative values were obtained for the clivus, including longitudinal relaxation time (T1, ms) and transverse relaxation times (T2 and T2*, ms). Segmentation was performed using ITK-SNAP software (version 3.8.0). For qMRI analysis, volumes of interest (VOIs) were manually delineated on the T1-weighted imaging (T1WI) sequence to include: clival invasion (excluding blood vessels or cystic areas identified on T1WI) and normal cervical vertebrae (autologous bone control). The VOIs were then applied to the corresponding slices of T1, T2, and T2* mapping sequences. Mean values were automatically calculated across all voxels within the VOIs. All VOI segmentations were performed by two independent radiologists with 15 and 4 years of experience in head and neck imaging, respectively. Before formal segmentation, both readers underwent a standardized preliminary training session using 5 separate NPC cases to standardize the definition of clival boundaries and VOI delineation procedures. Both readers were blinded to the patients’ clinical characteristics, imaging time points, and therapeutic efficacy outcomes. Prior to analyzing the entire cohort, measurement reproducibility was assessed in a randomly selected subset of 20 cases using intraclass correlation coefficient (ICC) for inter- and intra-observer reliability, along with Dice similarity coefficient (DSC) for spatial agreement in VOI delineation. Discrepancies in VOI placement were resolved by consensus between the two radiologists. After three months, all patients underwent repeat delineation by Radiologist 1 to assess intra-observer agreement. The values of ΔT1, ΔT2, and ΔT2* were respectively equivalent to the T1, T2, and T2* values of the clivus minus those of the normal cervical vertebrae.

### Statistical analysis

Statistical analyses were performed using IBM SPSS Statistics software (version 25.0, Chicago, IL) and R software (version 4.3.1). Mean values of T1, T2, and T2* mapping parameters within the VOIs were used as qMRI variables. The ICC values were interpreted as follows: poor inter-rater agreement < 0.40, fair = 0.40–0.59, good = 0.60–0.74, and excellent = 0.75–1.00. Statistical significance was set at p < 0.05 (two-tailed). Normally distributed data were presented as mean ± standard deviation (SD), while non-normally distributed data were presented as median with interquartile range (IQR). Categorical data are presented as absolute numbers. SyMRI parameters were compared between clival invasion and cervical vertebrae using the Paired t-test or Paired samples Wilcoxon signed rank test, as appropriate. Generalized estimating equation (GEE) was used to compare parameter values across time points. When significant overall differences were observed, pairwise comparisons with Bonferroni adjustment were performed. To evaluate the capability of qMRI metrics in predicting short-term therapeutic efficacy, the relative change (RC) for each parameter was calculated using the following formula:


$${\text{RC}}_{\text{parameter}}\left(\%\right)=\frac{\left({\text{Parameter}}_{\text{baseline}}-{\text{ Parameter}}_{\text{post-RT}}\right)}{{\text{Parameter}}_{\text{baseline}}}\times 100\%$$

Subsequently, receiver operating characteristic (ROC) curve analyses were performed to assess the discriminative ability of these longitudinal changes in predicting therapeutic response. Diagnostic performance was quantified using the area under the curve (AUC) with its 95% confidence interval. A multiparametric combined model incorporating RC_T1_, RC_T2_, and RC_T2*_ was constructed to evaluate whether the integration of multiple qMRI parameters could improve predictive performance. Internal validation using 1,000 bootstrap resamples was performed to reduce potential bias related to the small sample size and to assess the internal robustness of the predictive model.

## Results

### Participant characteristics

**Figure 1:**
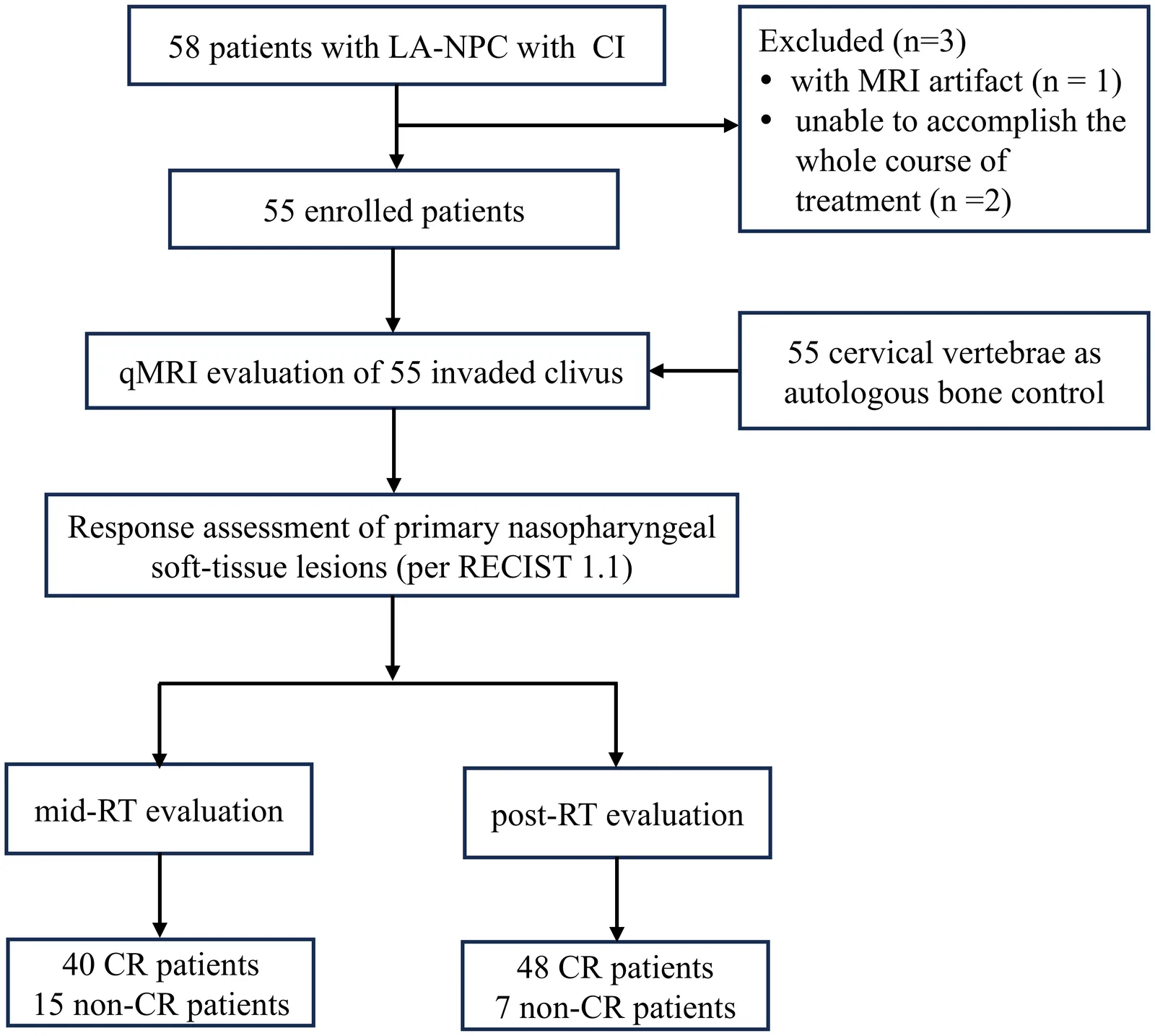
Flowchart of participants eligible for analysis.

**Figure 2:**
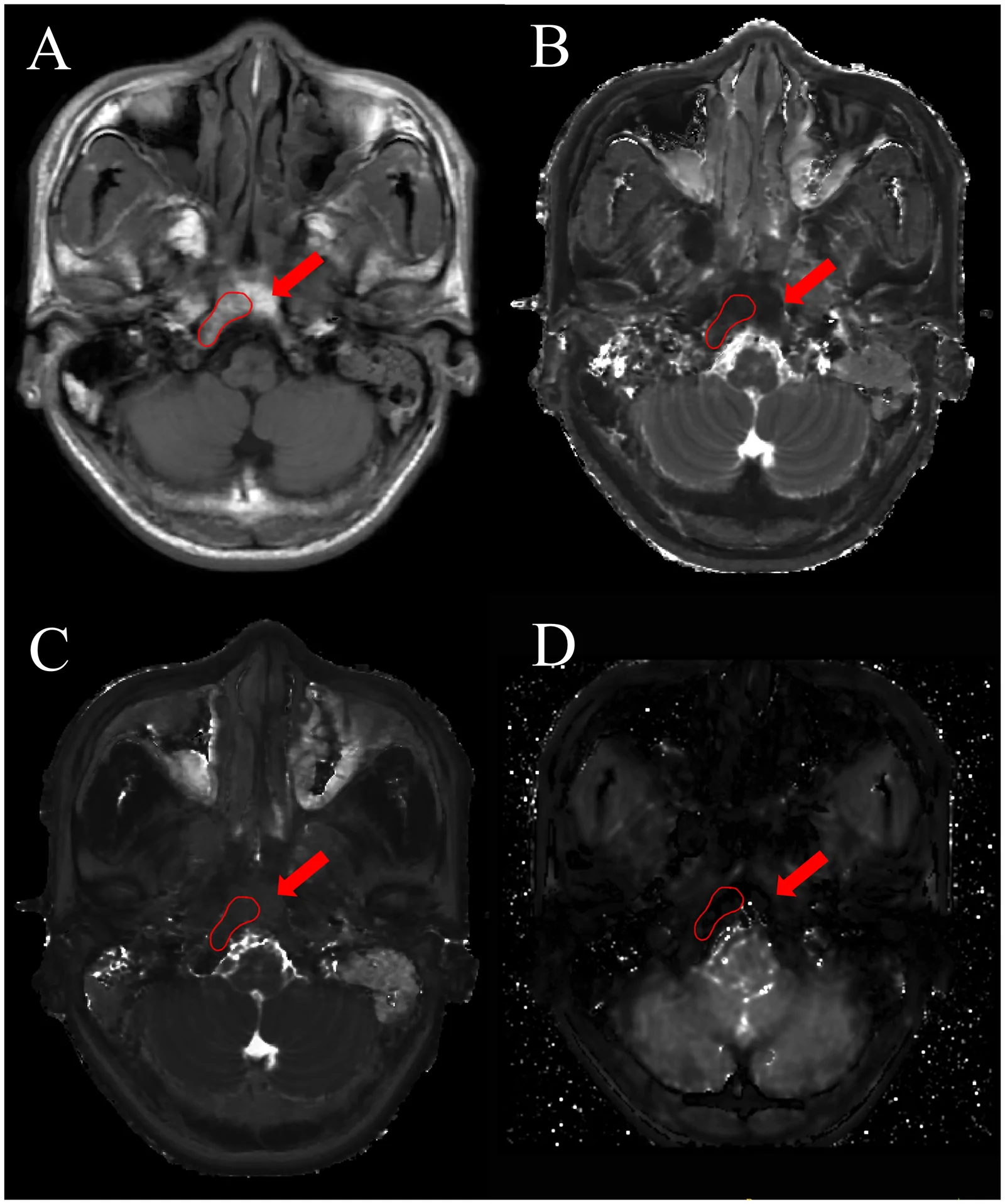
Representative images of clival invasion in a 34-year-old man with locally advanced nasopharyngeal carcinoma. The volume of interest (VOI; red outline) was manually delineated on conventional T1-weighted imaging **(A)** to encompass the tumor lesion within the clivus (red arrow). The same VOI was subsequently copied and applied to the corresponding co-registered quantitative T1 map **(B)**, quantitative T2 map **(C)**, and quantitative T2* map **(D)**. Although lesion conspicuity was limited on the T2* map **(D)**, likely because of susceptibility-related artifacts at the skull base, the co-registered VOI enabled consistent quantitative parameter extraction across all maps.

Among the total 58 patients, 3 patients were excluded because of interruption of treatment (n = 2) or poor image quality caused by motion artifacts (n = 1). Finally, a total of 55 patients meeting the inclusion criteria were enrolled. The patient selection process is summarized in **Figure 1**. The demographics of the patients are shown in **Table 1**, and representative imaging examples are shown in **Figure 2**.

**Table 1:** Clinical characteristics of 55 LA-NPC patients with clival invasion.

Characteristics	n=55
Median age (range)-yr	46(18-67)
Gender	
Male	44 (80.0%)
Female	11 (20.0%)
^#^Overall stage	
III	16 (29.1%)
IVA	39 (70.9%)
^#^T stage	
T3	26 (47.3%)
T4	29 (52.7%)

^#^ TNM staging followed the 8th edition of the AJCC staging system.

### Baseline qMRI parameters between clival invasion and cervical vertebrae

The T1, T2, and T2* values of clival invasion were 888.59 ± 302.69 ms, 91.02 ± 11.36 ms, and 7.72 ± 3.61 ms, respectively. The T1, T2, and T2* values of normal cervical vertebrae were 580.29 ± 53.26 ms, 81.76 ± 9.43 ms, and 5.59 ± 2.63 ms, respectively. The quantitative parameters of CI demonstrated significantly higher T1, T2, and T2* values compared to the normal cervical vertebrae (*p* < 0.001; **Table 2**). In clival invasion, T1 and T2* mapping values showed a significant decreasing trend throughout treatment, while T2 mapping values exhibited an increasing trend (*p* < 0.001; **Table 3**). The ICC values for T1, T2, and T2* measurements demonstrated excellent reliability, and the DSC values were 0.820 and 0.825 for delineation of the clival invasion and cervical vertebrae, respectively (**Supplementary Table 1**).

**Table 2:** T1, T2, and T2* mapping values between clival invasion and normal cervical vertebrae (n=55) at baseline.

Parameters	Clival invasion	Cervical vertebrae	Cohen’s d	T value	P value
T1 (ms)	888.59 ± 302.69	580.29 ± 53.26	1.033	7.663**<0.001**^#^	
T2 (ms)	91.02 ± 11.36	81.76 ± 9.43	0.666	4.942**<0.001**^#^	
T2*(ms)	7.72 ± 3.61	5.59 ± 2.63	0.558	4.142**<0.001**^#^	

^#^ Statistically significant.

^#^Values in bold indicate statistic significance.

**Table 3:** qMRI values of clival invasion.

Parameters	Baseline	Pre-RT	Mid-RT	Post-RT	Waldχ^2^	P value
T1 (ms)	821.41 (520.55)	706.26 (441.97)	618.37 (258.57)	588.30 (192.08)	27.977	**<0.001** ^#^
T2 (ms)	90.27 (18.67)	89.52 (18.27)	98.82 (10.62)	98.47 (11.58)	68.405	**<0.001** ^#^
T2* (ms)	7.49 (5.54)	5.80 (4.19)	5.04 (4.10)	4.96 (3.55)	20.479	**<0.001** ^#^

^#^ Statistically significant.

^#^Values in bold indicate statistic significance.

### The changes of qMRI parameters during the treatment

**Figure 3:**
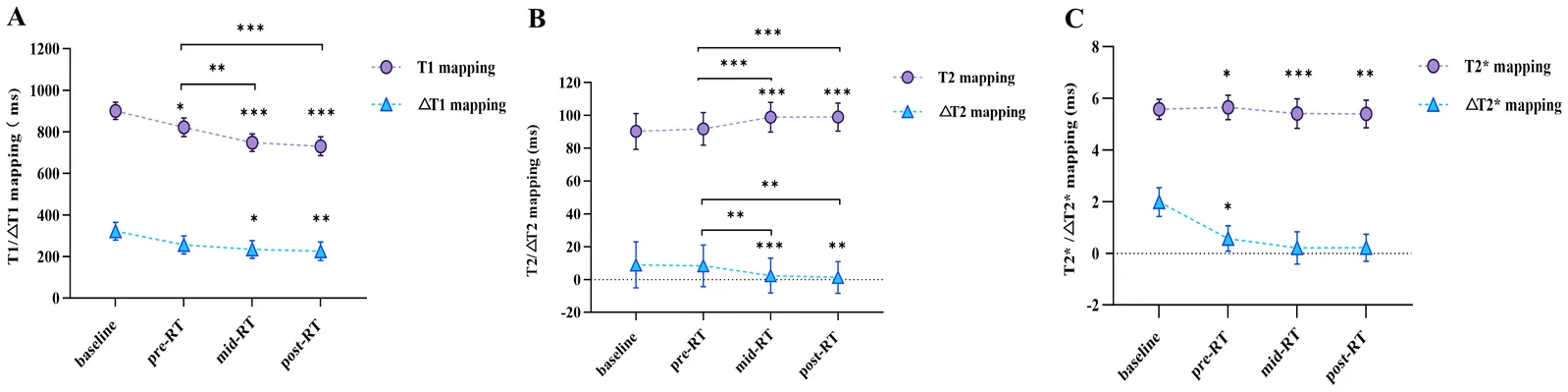
T1, T2, and T2* mapping and their corresponding ΔT1, ΔT2, and ΔT2* values in the CR group. T1 mapping and ΔT1 mapping **(A)**. T2 mapping and ΔT2 mapping **(B)**. T2* mapping and ΔT2* mapping **(C)**. (*) on the plots represent compared to the baseline, respectively. (*) on the lines represent the comparison of two groups connected with a line. * represents P value<0.05, ** represents P value <0.01, *** represents P value <0.001.

Patients were stratified into CR and non-CR groups based on the RECIST 1.1-defined response of the primary nasopharyngeal soft-tissue lesion. Longitudinal qMRI parameters of the clival invasion were then compared between these groups to explore whether clival qMRI changes differed according to primary soft-tissue treatment response. At mid-RT, 40 patients (72.7%) achieved CR in nasopharyngeal soft tissue tumor lesions, while 15 patients (27.3%) did not. At post-RT, 48 patients (87.3%) achieved CR in nasopharyngeal soft tissue tumor lesions, while 7 patients (12.7%) did not. As shown in **Table 4**, no statistically significant changes were observed for T1 and T2 mapping in the non-CR group (*p* > 0.05). Nevertheless, a statistically significant difference in T2* mapping been demonstrated (*p* < 0.001). Conversely, the CR group exhibited significant changes in all three parameters after treatment (*p* < 0.05; **Figure 3**; **Supplementary Table 2**). In the CR group, further time-point comparisons revealed statistically significant differences in T1 and T2* mapping values between t0 and t1, t2, and t3, while T2 mapping values showed statistically significant differences only between t0 and t2, and t3. In the comparison between t1 and t3, T1 and T2 mapping values showed significant differences, while T2* mapping values showed no significant difference. Notably, there were no significant differences in T1, T2, and T2* mapping values between t2 and t3 in the CR group (*p* > 0.05). Specific results are shown in **Figure 3**. and **Supplementary Table 3**.

**Table 4:** qMRI values of clival invasion in the non-CR group.

Parameters	Baseline	Pre-RT	Mid-RT	Post-RT	Waldχ^2^	P value
T1(ms)	657.71 (580.49)	529.06 (561.99)	588.84 (338.89)	603.54 (204.74)	0.804	0.848
T2(ms)	97.78 (29.10)	96.49 (35.37)	98.10 (14.40)	98.20 (16.09)	4.475	0.215
T2*(ms)	7.59 (5.53)	9.63 (7.59)	4.51 (5.49)	5.88 (7.54)	40.102	**<0.001** ^#^

^#^ Statistically significant.

^#^Values in bold indicate statistic significance.

### Relationship between regression of clival invasion and primary nasopharyngeal soft tissue tumor

To further investigate the relationship between the regression of clival invasion and primary nasopharyngeal soft tissue tumors, the differences in qMRI parameters (ΔT1, ΔT2, and ΔT2* mapping) were compared at each time point. The results showed that as treatment progressed, ΔT1, ΔT2, and ΔT2* values in the CR group gradually decreased during treatment (**Figure 3**). Specifically, the difference in ΔT2* mapping values between t0 and t1 was statistically significant, while pairwise comparisons at all other time points showed no significant differences. Notably, the differences in ΔT1 and ΔT2 mapping values between t0 and t1, and between t2 and t3 were all not statistically significant. However, a significant difference was observed between t1 and t3 for ΔT2 (*p* < 0.01; **Figure 3**). In contrast, in the non-CR group, no significant differences were observed in the changes of ΔT1 mapping values during treatment (*p* = 0.738), whereas both ΔT2 and ΔT2* values demonstrated significant changes (*p* < 0.001; **Supplementary Table 4**).

### Predictive performance of quantitative metrics for therapeutic response

**Figure 4:**
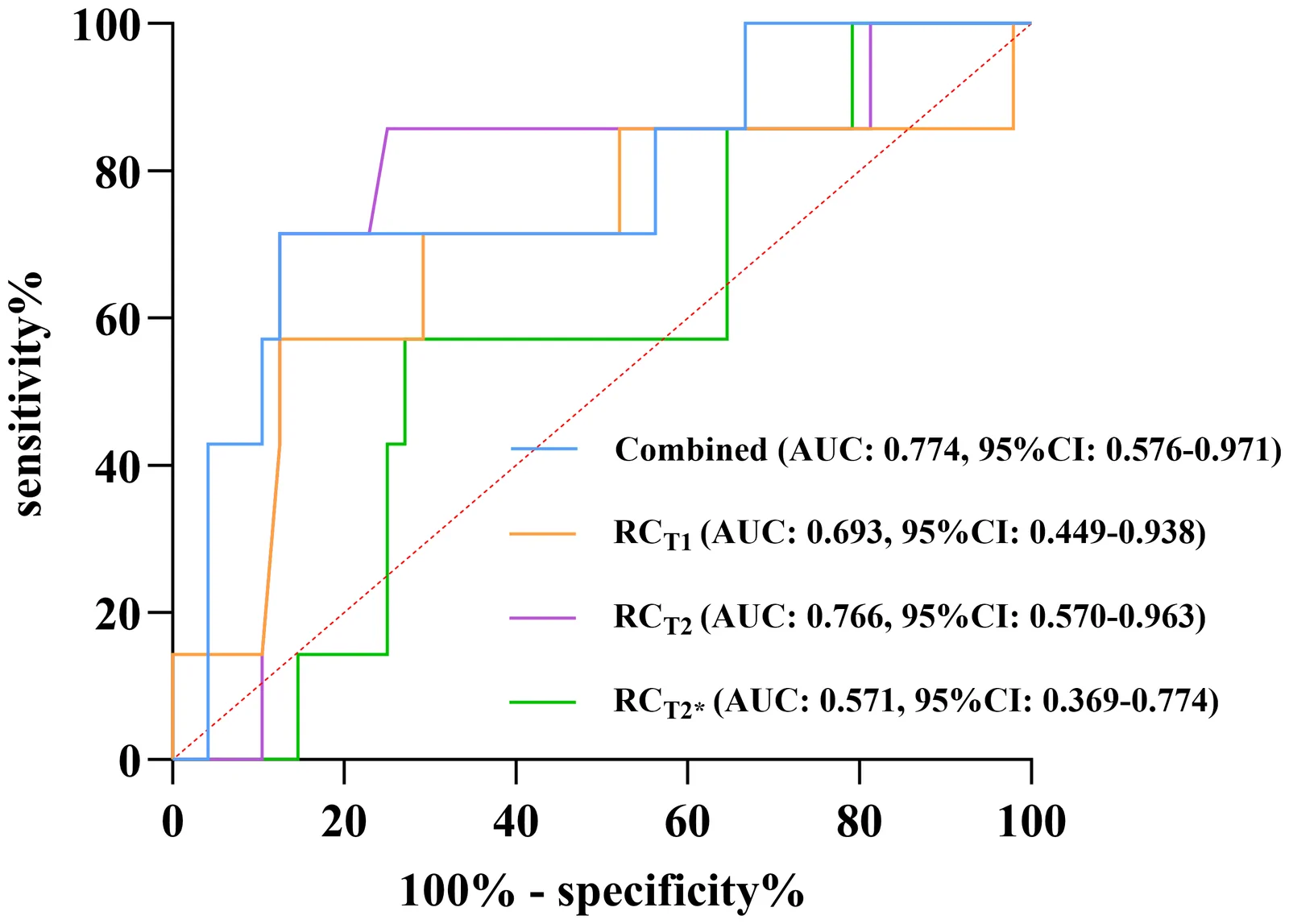
Receiver operating characteristic (ROC) curves of quantitative MRI parameter relative changes (RC_T1_, RC_T2_, and RC_T2*_) and the combined model for predicting treatment response.

ROC analysis was performed to evaluate the ability of longitudinal qMRI changes to distinguish between the CR and non-CR groups, and the results are shown in **Figure 4**. Among the evaluated parameters, only RC_T2_ and the multiparametric combined model incorporating RC_T1_, RC_T2_, and RC_T2*_ demonstrated statistically significant predictive performance (p<0.05). The multiparametric combined model achieved an AUC of 0.774 (95% CI: 0.576–0.971), while RC_T2_ alone showed a comparable AUC of 0.766 (95% CI: 0.570-0.963). Bootstrap-based pairwise comparisons indicated that the predictive performance of RC_T2_ was not significantly inferior to that of the combined model (p>0.05). In contrast, RC_T1_ (AUC = 0.693) and RC_T2*_ (AUC = 0.571) showed relatively lower predictive performance and did not provide additional predictive value over the reference models. To evaluate statistical robustness and reduce potential overestimation related to small-sample bias, internal validation was performed using 1,000 bootstrap resamples. Regarding model calibration, the bootstrap-derived results demonstrated good fitting accuracy, with a mean absolute error of 0.039 and a 0.9 quantile of absolute error of 0.082.

## Discussion

Clival invasion occurs in nearly 50% of recurrent nasopharyngeal carcinoma cases (3, 7). However, the RECIST criteria consider only soft-tissue lesions as target lesions for baseline measurement and therapeutic response evaluation, neglecting osseous involvement (11). While conventional MRI readily identifies soft-tissue regression, assessing CI regression remains difficult due to complex bone signal changes. Persistent tumor-like enhancement may occur in bone even without viable tumor cells, a phenomenon reported in up to 60% of NPC patients by the fourth year of follow-up (26). These alterations result from scar tissue gradually replacing the infiltrated skull base after radiotherapy. Enhancement may be present in the early, immature phase but is generally absent in mature scar tissue. Additionally, new bone marrow changes after radiotherapy may stem from radiation osteitis, osteoradionecrosis, or benign post-treatment alterations (27, 28), complicating the determination of local complete remission and potentially affecting subsequent adjuvant therapy decisions.

Although conventional functional imaging techniques, such as diffusion-weighted imaging (DWI), are widely adopted in clinical and research settings, they are prone to susceptibility artifacts that may introduce measurement errors in head and neck malignancies (29). Furthermore, while PET/CT offers excellent metabolic characterization, its utility for repeated longitudinal monitoring is limited by ionizing radiation, high cost, and a known risk of false-positive findings driven by radiation-induced inflammation (11). In this context, the potential incremental value of qMRI lies in its ability to translate subjective signal intensity changes into objective and reproducible quantitative metrics. Thus, qMRI may serve as a promising non-invasive approach for evaluating CI and monitoring treatment response in LA-NPC.

Our results showed significantly higher T1, T2, and T2* values in the clival invasion compared to the cervical vertebrae at baseline. The increase in T1 aligns with prior reports (23) and may reflect tumor necrosis releasing extracellular macromolecules (22, 30). Although T2 mapping was recognized as a reliable biomarker of tissue water content and typically decreased in soft-tissue tumors (31, 32), our results contradict this. This discrepancy may stem from inherent compositional differences between bone and soft tissue. Tumor-induced disruption of the bone surface permits water influx into the matrix, while cancer cell infiltration into the marrow cavity induces edema, counteracting the expected reduction in free water content due to cellular proliferation. Consequently, T2 mapping values increase in tumor-invaded bone lesions—a finding consistent with observations from bone metastasis studies that T2 mapping values in active bone metastatic lesions are higher than those in inactive lesions (19). T2* mapping can detect deoxyhemoglobin content in tissues. The unlimited proliferation of tumor cells leads to insufficient tissue blood supply and frequently induces a hypoxic microenvironment, thereby resulting in increased deoxyhemoglobin content compared to normal tissues. Owing to its strong paramagnetic properties, deoxyhemoglobin typically causes a reduction in T2* mapping values (17). However, we observed higher T2* mapping values in CI, consistent with previous studies (33, 34). This may be attributed to magnetic field inhomogeneity caused by the sensitivity differences of T2* mapping to bone trabeculae and bone marrow interfaces, which leads to lower T2* mapping values in bone tissue (17, 35). Tumor cells disrupt the normal bone trabecular structure, reducing magnetic field inhomogeneity and potentially offsetting the effect of hypoxic hemoglobin (36, 37). As a result, the net effect is higher T2* mapping values in CI relative to normal bone.

With treatment, T1 and T2* mapping values exhibited a gradual decrease in the clival invasion of the CR group. The decline in T1 values likely reflects reduced tumor burden and decreased levels of necrotic material and cellular macromolecules. The decrease in T2* may be attributed to the restoration of bone trabecular structure, which increases magnetic field inhomogeneity and enhances the paramagnetic effect of marrow fat. Conversely, T2 values showed a gradual increase in the CR group, consistent with reduced cellularity and a relative rise in extracellular free water after tumor regression. In the non-CR group, T1 and T2 values showed no significant change, consistent with persistent tumor activity due to treatment insensitivity or resistance, although T2* also decreased significantly. These findings preliminarily indicate that T1 and T2 mapping may serve as more reliable biomarkers than T2* for evaluating clival response, and support a correlation between clival parameter normalization and regression of nasopharyngeal soft tissue lesions. This suggests that complete remission of the primary soft tissue tumor may be associated with a higher likelihood of clival remission. Indeed, T2* mapping was hampered by limited lesion conspicuity, likely owing to susceptibility-related artifacts at the skull base, whereas T1 and T2 mapping provided more adequate anatomical delineation and robust quantitative values from the same co-registered region. These observations suggest that T1 and T2 parameters derived from the MAGiC acquisition may offer more dependable clinical value for characterizing clival invasion. Further optimization of T2* acquisition and post-processing strategies is warranted to improve its reliability in this anatomically challenging region.

We observed that in the CR group, the difference values (ΔT1, ΔT2, ΔT2*) between the clival invasion and the cervical vertebrae of the patient gradually decreased during treatment, indicating that the tumor in the clival invasion gradually regressed, and the three values were close to 0 at the end of treatment. Among these, ΔT1 and ΔT2 values exhibited significant changes before and after radiotherapy, but no statistically significant differences were observed before and after IC. Conversely, ΔT2* values showed statistically significant change before and after IC. In the non-CR group, there were significant differences between ΔT2 and ΔT2* values. Further analysis revealed that the change in ΔT2 values was more pronounced after radiotherapy than after IC, with the former difference being statistically significant. This indicates that T2 mapping may be more sensitive in monitoring bone changes caused by radiotherapy and is more likely to be affected by bone marrow edema, potentially masking the true trend of ΔT2 mapping changes in the non-CR group. Additionally, we found that the ΔT2* mapping values in the CR group showed no significant difference when comparing t0 with t2 and t3 time points, respectively, while the T2* values showed differences in pairwise comparisons at the aforementioned time points. This may be because the ΔT2* values approach zero near the end or at the completion of radiotherapy, making them more susceptible to interference from non-original data due to the small parameter value, as well as the conservatism of Bonferroni correction. Nevertheless, the decrease in the ΔT2* value can reflect the gradual recovery of the clival invasion towards near-normal cervical vertebrae after treatment.

In our receiver operating characteristic (ROC) curve analysis, the relative change in T2 values (RC_T2_) and the combined multiparametric model demonstrated robust predictive efficacy. Notably, these quantitative metrics revealed a synchronous trajectory between the alterations in clival osseous parameters and the regression of soft-tissue lesions. This synchrony corroborates our longitudinal observation that, as treatment progresses, the qMRI parameters of the clival invasion in the complete response (CR) group gradually normalize, closely approaching the baseline values of unaffected cervical vertebrae. These dynamic shifts indicate that qMRI parameters can reliably monitor intra-therapeutic osseous remodeling, thereby offering a novel quantitative paradigm for the longitudinal evaluation of skull base involvement. Furthermore, the predictive performance of the combined model did not show a statistically significant improvement over RC_T2_ alone. This lack of significance implies that the single RC_T2_ parameter inherently possesses excellent predictive efficacy for therapeutic response. However, given the limited sample size in the current study, these preliminary findings necessitate further validation through larger, prospective cohorts to confirm the standalone diagnostic value of RC_T2_ versus combined multiparametric models.

In the intensity-modulated radiation therapy (IMRT) era, a standard 70 Gy radiation dose for NPC can achieve local control rates exceeding 90% (1). However, the associated radiation side effects can significantly diminish patients’ quality of life, leading to debate about the necessity of a uniform 70 Gy dose for all NPC patients. Research from the two-dimensional radiotherapy era indicated that 60 Gy, when combined with IC and CCRT, could achieve favorable local control (38). Subsequent studies suggest that selected low-risk NPC patients receiving approximately 60 Gy via IMRT attain comparable local control with reduced toxicity, yielding outcomes similar to those with 70 Gy (39, 40). Based on this evidence, we focused on the 60 Gy time point (t2) for further analysis. Our results revealed no significant differences in T1, T2, and T2* values, nor in their parameter differences (ΔT1, ΔT2, and ΔT2*), between the time points following 60 Gy (t2) and completion of radiotherapy (t3) in the CR group. This statistical plateau suggests that biophysical stabilization of clival invasion may occur by 60 Gy, in parallel with the macroscopic regression of nasopharyngeal soft-tissue lesions. Thus, for treatment-sensitive patients who achieve soft-tissue CR at 60 Gy, these exploratory findings provide an imaging-based rationale for future clinical trials to investigate whether the conventional 70 Gy regimen could be safely tailored. Ultimately, this highlights the potential of qMRI to assist in early risk stratification of LA-NPC patients presenting with skull base invasion.

Several limitations warrant consideration. First, although this was an exploratory study, the generalizability of our findings is limited by the single-center design, relatively small sample size, lack of long-term follow-up, and absence of an independent external validation cohort. Although internal validation was performed, future multicenter prospective studies with larger cohorts and long-term outcome data are essential to confirm these results in an independent external setting. Second, this study did not include a direct intra-individual comparison between qMRI parameters and conventional MRI or PET/CT metrics. Therefore, the incremental value of qMRI over standard imaging modalities cannot be definitively established based on the present findings. Future studies should include direct comparisons with standard MRI and PET/CT metrics to further validate the added clinical value of qMRI. Third, the absence of histopathological confirmation in evaluating the response of the clival invasion during treatment required us to rely on comprehensive clinical and imaging data for diagnosis. While this approach is standard practice, it introduces a degree of uncertainty. Finally, the manual delineation of VOIs may have been influenced by the relatively thick slice thickness used in imaging. To mitigate this, multiple delineations were performed to improve accuracy.

## Conclusion

In conclusion, our study demonstrates that qMRI-derived T1, T2, and T2* mapping holds promise as quantitative imaging biomarkers for assessing the treatment response of clival invasion in LA-NPC. The positive correlation between clival parametric alterations and primary soft-tissue tumor regression underscores the potential utility of these metrics in monitoring early local remission. Although preliminary, these findings provide non-invasive evidence that could inform future studies exploring individualized treatment strategies in this patient population.

## Data Availability

The raw data supporting the conclusions of this article will be made available by the authors, without undue reservation.
