# Supplementary material for: Quantitative MRI in the assessment of clival invasion in locally advanced nasopharyngeal carcinoma
Source: Front Oncol. 2026 Aug 27;16:1751817. doi: 10.3389/fonc.2026.1751817 (PMC13558114; doi:10.3389/fonc.2026.1751817)
Supplement: Supplementary file 1 [file Table1.docx]

**Supplementary Table 1.** Inter- and intra-observer reproducibility of qMRI measurements and VOI delineation.

| Parameters  (ms) | Intraclass correlation coefficients (95% CI) | | | | | Dice similarity coefficient (95% CI) | |
| --- | --- | --- | --- | --- | --- | --- | --- |
|  | Intra-observer Inter-observer | | | | | Clival invasion | Cervical vertebrae |
|  | Clival invasion | Cervical vertebrae | Clival invasion | Cervical vertebrae | |  |  |
| T1 | 0.988(0.977-0.994) | 0.944(0.895,0.970) | 0.993 (0.986-0.996) | | 0.975 (0.954-0.987) | 0.820 (0.803-0.837) | 0.825 (0.791-0.859) |
| T2 | 0.874(0.773-0.932) | 0.858(0.748-0.922) | 0.954 (0.915-0.975) | | 0.950 (0.898-0.974) |  |  |
| T2* | 0.771(0.609-0.872) | 0.757(0.560-0.868) | 0.830 (0.703-0.906) | | 0.855 (0.742-0.920) |  |  |

CI: confidence interval.
