# Supplementary material for: Quantitative MRI in the assessment of clival invasion in locally advanced nasopharyngeal carcinoma
Source: Front Oncol. 2026 Aug 27;16:1751817. doi: 10.3389/fonc.2026.1751817 (PMC13558114; doi:10.3389/fonc.2026.1751817)
Supplement: Supplementary file 2 [file Table2.docx]

**Supplementary Table 2.** qMRI values of clival invasion in the CR group (n=48).

| parameters | baseline | pre-RT | mid-RT | post-RT | Waldχ^2^ | P value |
| --- | --- | --- | --- | --- | --- | --- |
| T1 (ms) | 841.30(521.31) | 707.34(450.92) | 631.17(244.70) | 587.61(187.54) | 33.750 | **<0.001^#^** |
| T2 (ms) | 89.83(18.25) | 89.30(17.52) | 98.95(10.80) | 99.79(12.10) | 71.684 | **<0.001^#^** |
| T2* (ms) | 7.27(5.28) | 5.57(4.31) | 5.09(4.05) | 4.86(3.48) | 16.902 | **0.001^#^** |

^#^ Statistically significant
