# Supplementary material for: Quantitative MRI in the assessment of clival invasion in locally advanced nasopharyngeal carcinoma
Source: Front Oncol. 2026 Aug 27;16:1751817. doi: 10.3389/fonc.2026.1751817 (PMC13558114; doi:10.3389/fonc.2026.1751817)
Supplement: Supplementary file 3 [file Table3.docx]

**Supplementary Table 3.** Pairwise comparisons of quantitative MRI parameters across different time points.

| parameter/group | baseline vs. pre-RT | | baseline vs. mid-RT | | baseline vs. post-RT | | | mid-RT vs. post-RT | |
| --- | --- | --- | --- | --- | --- | --- | --- | --- | --- |
|  | Cohen's d | P value (95% CI) | Cohen's d | P value (95% CI) | Cohen's d | | P value (95% CI) | Cohen's d | P value (95% CI) |
| **T1 (ms)** |  |  |  |  |  |  | |  |  |
| Total (n=55) | 0.551 | **<0.001^#^** (0.268, 0.835) | 0.699 | **<0.001^#^** (0.404, 0.994) | 0.708 | **<0.001^#^** (0.412, 1.003) | | 0.211 | 0.705 (-0.056, 0.478) |
| CR (n=48) | 0.437 | **0.015^#^** (0.141, 0.733) | 0.760 | **<0.001^#^** (0.438,1.081) | 0.811 | **<0.001^#^** (0.485, 1.137) | | 0.237 | 0.602 (-0.050, 0.524) |
| non-CR (n=7) | NA |  |  |  |  |  | |  |  |
| **T2 (ms)** |  |  |  |  |  |  | |  |  |
| Total (n=55) | -0.110 | 1.000 (-0.375, 0.155) | -0.889 | **<0.001^#^** (-1.202, -0.577) | -0.944 | **<0.001^#^** (-1.262, -0.626) | | -0.003 | 1.000 (-0.267, 0.262) |
| CR (n=48) | -0.170 | 1.000 (-0.455, 0.115) | -0.979 | **<0.001^#^** (-1.323, -0.635) | -1.046 | **<0.001^#^** (-1.398, -0.694) | | -0.013 | 1.000 (-0.296, 0.270) |
| non-CR (n=7) | NA |  |  |  |  |  | |  |  |
| **T2* (ms)** |  |  |  |  |  |  | |  |  |
| Total (n=55) | 0.390 | **0.023^#^** (0.116, 0.664) | 0.640 | **<0.001^#^** (0.316,0.891) | 0.481 | **0.002^#^** (0.202, 0.760) | | -0.043 | 1.000 (-0.307, 0.222) |
| CR (n=48) | 0.421 | **0.021^#^** (0.126, 0.717) | 0.574 | **<0.001^#^** (-0.879, -0.269) | 0.476 | **0.006^#^** (0.178, 0.775) | | 0.002 | 1.000 (-0.280, 0.285) |
| non-CR (n=7) | -0.082 | 1.000 (-0.824, 0.660) | 0.953 | 0.070 (-0.060, 1.847) | 0.603 | 0.663 (-0.202, 1.409) | | -0.282 | 1.000 (-1.037, 0.473) |

^#^ Statistically significant. NA: Post-hoc pairwise comparisons were not performed for parameters with an overall P > 0.05.
