# Supplementary material for: Quantitative MRI in the assessment of clival invasion in locally advanced nasopharyngeal carcinoma
Source: Front Oncol. 2026 Aug 27;16:1751817. doi: 10.3389/fonc.2026.1751817 (PMC13558114; doi:10.3389/fonc.2026.1751817)
Supplement: Supplementary file 4 [file Table4.docx]

**Supplementary Table 4.** Post-treatment differences between clival invasion and normal cervical vertebrae in the CR and non-CR groups.

| parameters | | CR group (n=48) | | | | | non-CR group (n=7) | | | | | | | | |
| --- | --- | --- | --- | --- | --- | --- | --- | --- | --- | --- | --- | --- | --- | --- | --- |
| (ms) | baseline | | pre-RT | mid-RT | post-RT | P | | baseline | | pre-RT | mid-RT | post-RT | | | P |
| ΔT1 | 300.19  (531.15) | | 152.48  (377.70) | 98.88  (229.07) | 104.82  (184.77) | **0.014^#^** | | | 86.98  (692.42) | 55.19  (539.98) | 83.41  (583.10) | 60.99  (500.15) | | 0.738 | |
| ΔT2 | 8.99  (18.41) | | 9.80  (17.82) | 0.99  (11.50) | -0.57  (12.55) | **<0.001^#^** | | | 19.67  (23.32) | 5.15  (40.93) | -0.95  (27.69) | 1.51  (32.73) | **<0.001^#^** | | |
| ΔT2* | 0.74  (4.96) | | 0.69  (3.80) | 0.61  (4.18) | 0.35  (4.81) | **0.017^#^** | | 2.45  (6.25) | | 6.42  (8.87) | 4.47  (8.59) | 0.94  (13.57) | **<0.001^#^** | | |

^#^ Statistically significant
